# Linking soil microbial genomic features to forest-to-pasture conversion in the Amazon

**DOI:** 10.1128/spectrum.01561-24

**Published:** 2025-03-05

**Authors:** Andressa M. Venturini, Júlia B. Gontijo, Louis Berrios, Jorge L. Mazza Rodrigues, Kabir G. Peay, Siu M. Tsai

**Affiliations:** 1 Cell and Molecular Biology Laboratory, Center for Nuclear Energy in Agriculture, University of São Paulo, Piracicaba, São Paulo, Brazil; 2 Department of Biology, Stanford University, Stanford, California, USA; 3 Department of Environmental Science, American University, Washington, DC, USA; 4 Department of Land, Air, and Water Resources, University of California-Davis, Davis, California, USA; 5 Department of Earth System Science, Stanford University, Stanford, California, USA; Oklahoma State University, Stillwater, Oklahoma, USA

**Keywords:** Amazon rainforest, deforestation, land-use change, microbial ecology, soil microbiology, bacteria, archaea, metagenomics, metagenome-assembled genomes, genomic characteristics

## Abstract

Amazonian soil microbial communities are known to be altered by land-use change. However, attempts to understand these impacts have focused on broader community alterations or the response of specific microbial groups. Here, we recovered and characterized 69 soil bacterial and archaeal metagenome-assembled genomes (MAGs) from three forests and three pastures of the Eastern Brazilian Amazon and evaluated the impacts of land conversion on their genomic features. Pasture MAGs had significantly higher GC content (64.9% vs 60.2%), genome size (4.0 vs 3.1 Mbp), and number of coding sequences (4,058 vs 3,306) compared to forest genomes. Taxonomically, MAGs belonged to eight phyla; however, most (90%) had low similarity to previously known species, indicating potentially novel taxa at multiple levels. We also observed that the functional profiles associated with biogeochemical cycling and carbohydrate-active enzyme genes were impacted by forest conversion, with pasture MAGs exhibiting a notably higher number of both gene groups. Together, these data constitute the largest single-sourced genomic data set from upland soils of the Brazilian Amazon to date and increase the known MAG richness in these soils by 78%. Our data, therefore, not only add to a neglected yet emerging field but, importantly, highlight that land-use change has drastic impacts on the genomic characteristics and functional traits of dominant soil microbes.

**IMPORTANCE:**

The Brazilian Amazon is facing unprecedented threats, including increasing deforestation and degradation, which together impact half of the original forest area. Soil microorganisms are sensitive indicators of land-use change, linked to a rise in microbial methane emissions and antibiotic-resistance genes in the Amazon. However, most Amazonian soil microbes remain unknown, and little attention has been given to their genomes. Using sequencing and bioinformatics, we recovered and characterized 69 soil bacterial and archaeal genomes (metagenome-assembled genomes). These abundant members of the microbial communities diverged across forests and pastures in terms of taxonomic and functional traits. Forest conversion favors organisms with specific genomic features — increased GC content, genome size, and gene number — selecting for microorganisms that can thrive under altered conditions. Our paper helps us understand the intricate relationships between microbes and the environment, which are crucial pieces of information for comprehensive soil health assessments and future policy formulation.

## OBSERVATION

Home to more than half of bacterial and fungal species, soils are Earth’s most biodiverse habitat (1). Yet, this often-unknown diversity and its contribution to critical soil ecosystem services remain unclear. The recovery of metagenome-assembled genomes (MAGs) has expanded our knowledge of terrestrial microbes (2); however, studies on soil MAGs from tropical areas remain scarce despite the high diversity and importance of these ecosystems. For instance, only four studies have recuperated soil MAGs from Brazilian Amazon uplands — obtained through consortia cultivation (3) or direct DNA extraction from the field (4) and controlled experiments (5, 6), comprising a total of 88 genomes from 24 metagenomes. These studies collectively suggest that a large pool of novel taxa exists in these understudied areas of continental proportions, but their genomic features and functional traits have yet to be described and understood.

Understanding Amazonian soil microbial diversity and function has never been more urgent. The Amazon faces escalating pressure from anthropogenic actions, including land-use change and degradation that, combined, impact half of the original forest area (7, 8). Forest conversion alters the physical and chemical properties of Amazonian soils and their archaeal and bacterial communities, with significant implications for human health and greenhouse gas cycling (9). In particular, the transformation of these areas into pastures is now recognized to change methane microbial communities and create a sink-to-source shift in Amazonian soils (9, 10). Not surprisingly, a large number of studies have focused on land-use impacts at a community level using amplicon sequencing and read-based metagenomics (as reviewed in reference [10]). While useful for understanding the overall impacts of human perturbations, these approaches overlook genomic features crucial for assessing the relationship between microbes and their environment (11). Besides, to date, a large fraction of Amazonian soil microbial communities is still unknown to us (6).

**Fig 1:**
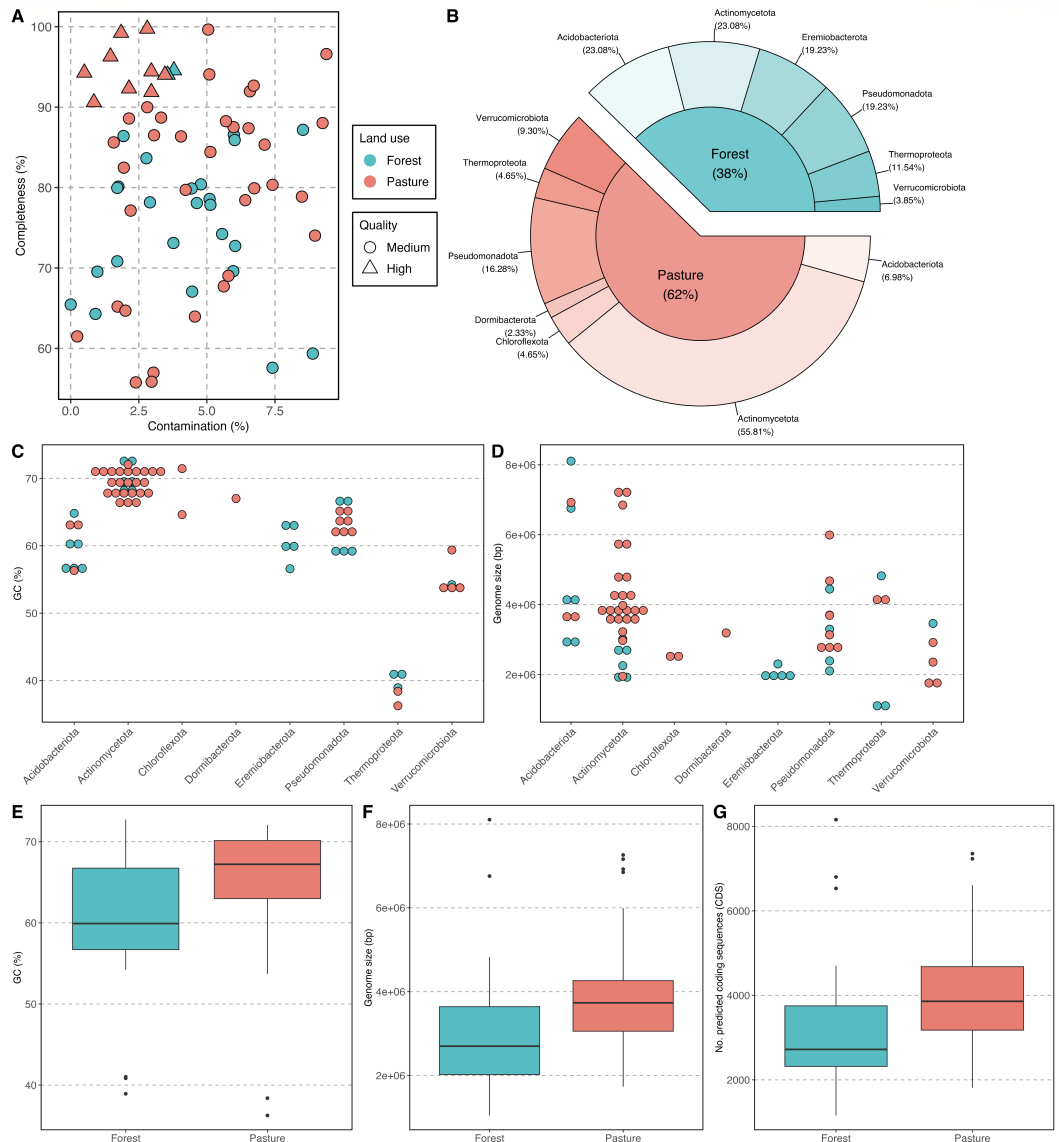
Summary of the MAGs found in forest and pasture soils. (**A**) Completeness (%) and contamination (%) of each medium- and high-quality MAG. (**B**) Taxonomic classification at the phylum level. (**C**) GC content (%) and (**D**) genome size (bp) distributed across phyla. (**E**) GC content (%), (**F**) genome size (bp), and (**G**) number of predicted coding sequences (CDSs) of forest and pasture MAGs. Pasture MAGs had higher GC content (*P* = 0.01), size (*P* = 0.004), and CDSs (*P* = 0.004) than forest MAGs.

Here, we used genome-based metagenomics to recover and characterize novel soil MAGs from the Amazon rainforest. We investigated how forest-to-pasture conversion impacts the genomic features and the taxonomic and functional traits of these most abundant microbial community members. From 36 soil metagenomes collected in three forests and three pastures of the Eastern Brazilian Amazon (State of Pará), we obtained 69 MAGs through read assembly and binning: 26 from forests and 43 from pastures (Supplemental text; Tables S1 to S5). Based on MIMAG standards (12), quality-controlled MAGs are medium- (≥50% completeness and <10% contamination) and high-quality drafts (>90% completeness and <5% contamination) (Fig. 1A). Overall, pasture MAGs had higher GC% (*P* = 0.01, mean of 64.9% vs 60.2%) and size (*P* = 0.004, mean of 4.0 vs 3.1 Mbp) than forest MAGs (Fig. 1E and F), but no significant correlation (*P* > 0.05) was found between both features. Together, these results not only represent the largest single genomic data set from upland soils of the Brazilian Amazon to date but also indicate genomic alterations of soil microbial communities in response to deforestation in the tropics.

GC content is highly influenced by the environment (13), and land conversion has been shown to reduce the abundance of short metagenomic sequences that are low-GC% (from 30% to 55%) in Western Amazonian soils (10). Considering the substantial methodological differences (read- vs genome-based metagenomics) and geographical distance between the sites of our studies (>1,000 km), this may represent a general microbial response to Amazonian forest-to-pasture conversion. Pastures in the Amazon are established and maintained through forest clearing and burning (9, 10), and results from coniferous forests have revealed that fire-impacted soil microbial communities have higher GC%, a trait linked to enhanced thermal stability (14). Pasture soils are also more exposed to higher temperatures and direct sunlight, and taxa resistant to UV irradiation (such as *Actinomycetota* members*,* which dominated our pasture communities [Fig. 1B]) seem to have a greater GC% than UV-sensitive microorganisms (15). Although not significant, predicted growth conditions also revealed that our pasture MAGs have higher minimum (22.7°C vs 22.1°C), optimal (36.3°C vs 35.6°C), and maximal (43.8°C vs 42.9°C) temperatures than those present in forests (Table S10). In addition, compared to aquatic and host-associated ecosystems, terrestrial microorganisms have larger genomes, reflecting the greater environmental fluctuations they experience (such as temperature changes) (16). This is also observed for ubiquitous taxa, which contain a higher proportion of genes linked to environmental adaptation (17). Thus, we hypothesize that generalist microorganisms with larger genomes may have an adaptive advantage in pasture soils. Interestingly, Wilhelm et al. (18) observed that soils with higher health are associated with smaller genome sizes.

Our MAGs spanned both bacterial (64 MAGs) and archaeal (5 MAGs) domains, representing 8 phyla and 13 classes (Fig. 1B through D; Table S6). *Actinomycetota* dominated pasture communities, while *Acidobacteriota* were reduced, as previously seen in other studies (10). *Eremiobacterota* were unique to forests, while *Chloroflexota* and *Dormibacterota* were found only in pastures. Amazonian forest soils are typically more acidic (9, 10), favoring the acid-tolerant phylum *Eremiobacterota*, with members that prefer soil pH below 6 (19), which was confirmed by our growth condition predictions (mean optimum pH = 5.4; Table S10). In fact, pasture MAGs have slightly higher pH requirements than forest genomes (not significant; optimum pH, 5.79 vs 5.75; maximum pH, 7.64 vs 7.39). *Chloroflexota* were also exclusively detected in Amazonian pasture soils under different moisture regimes (6), with the *Ktedonobacteria* class present across both studies. *Ktedonobacteria* possess high genome plasticity, enabling rapid adaptation to environmental shifts (20). Interestingly, all soil forest MAGs and most pasture MAGs could not be fully classified at the species, genus, and order levels (46, 15, and 1, respectively) by GTDB-Tk (release R08-RS214) (21), suggesting possible novel taxa. Average nucleotide identity (ANI) analysis, however, revealed that some of these genomes may belong to the same species (10 MAG pairs have an ANI ≥ 95% between themselves) (Table S7).

**Fig 2:**
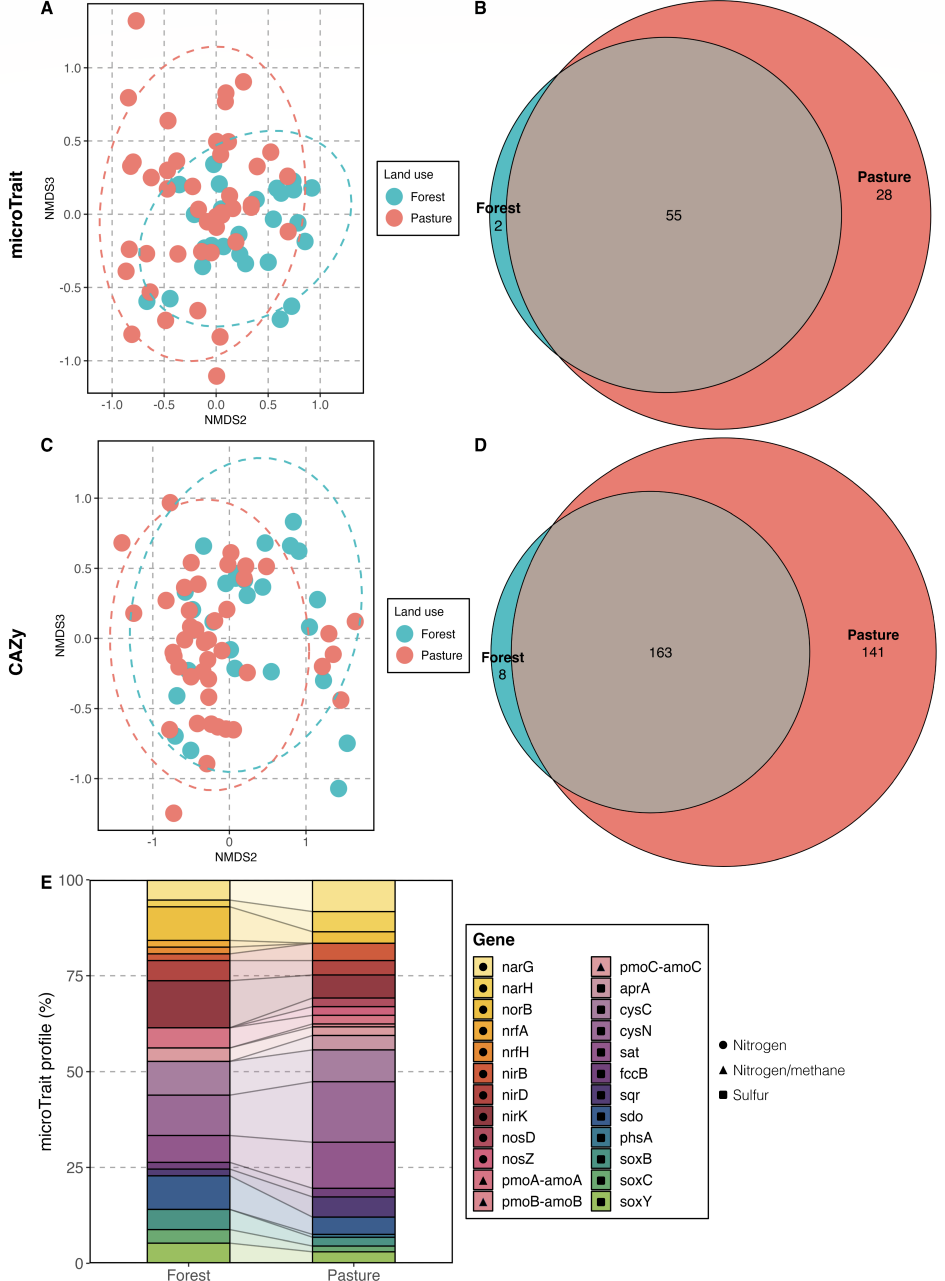
Genes found in forest and pasture soil MAGs using hidden Markov models. (**A**) Non-metric multidimensional scaling (NMDS) and (**B**) Venn diagram of biogeochemical cycling genes (microTrait). (**C**) NMDS and (**D**) Venn diagram of CAZy genes. (**E**) Profile of microTrait genes related to the nitrogen, methane, and sulfur cycles.

The observed variations in genome size across our forest and pasture sites can also influence gene diversity and functional versatility (22). Although no significant differences (*P* > 0.05) were observed for coding density (mean of 89.6% for pasture vs 88.2% for forest), the number of predicted coding sequences in pasture MAGs was significantly higher (*P* = 0.004, mean of 4,058 vs 3,306) than in forest genomes (Fig. 1G), a feature strongly correlated with genome size (*R* = 0.99, *P* < 0.001). Land-use change also impacted the functional traits of dominant soil microbes across land uses (PERMANOVA based on Jaccard distance), including genes related to biogeochemical cycling (microTrait, *F*_1,67_ = 2.079, *R*^2^ = 0.03, *P* = 0.007) (23) and carbohydrate-active enzyme families (CAZy, *F*_1,67_ = 2.164, *R*^2^ = 0.031, *P* = 0.004) (24) (Fig. 2; Tables S8 and S9). Pasture MAGs exhibited a notably higher number of both gene groups per MAG (microTrait, *P* = 0.005, mean of 21 vs 16; CAZy, *P* = 0.015, mean of 53 vs 38), traits also positively correlated with genome size (microTrait, *R* = 0.68, *P* < 0.001; CAZy, *R* = 0.6, *P* < 0.001). Similarly to our previous findings (6), several marker genes of critical biogeochemical processes were also found in MAGs, such as those related to the carbon, nitrogen, and sulfur cycles (Fig. 2E). Forests possess a higher relative abundance of organisms harboring denitrification-related genes, an association formerly discussed in references (10) and (25). Interestingly, although most MAGs were recovered from pastures, the genes *nrf*A and *nrf*H were uniquely found in forest genomes. These are associated with nitrite reduction in dissimilatory nitrate reduction to ammonium — an important nitrogen pathway in terrestrial ecosystems, which can decrease leaching losses and limit substrate availability for denitrification, thus reducing nitrous oxide emissions (26). Using a microarray (GeoChip) technology, Paula et al. (25) showed that the presence and abundance of the *nrf*A gene were highly correlated with primary forest soils in the Western Amazon.

Our findings provide much-needed data about the microbial diversity and functionality in Amazonian soils — an environment that plays critical roles in global change scenarios. These genomic insights, therefore, not only enhance our understanding of the intricate relationships within this ecosystem but also provide crucial knowledge for comprehensive soil health assessments as well as effective sustainable management and conservation practices.

## Data Availability

The raw, cleaned/filtered, and merged metagenomic sequences, as well as co-assemblies and sequences of the metagenome-assembled genomes, are available on the KBase platform at https://doi.org/10.25982/159671.262/2496635. High-quality metagenome-assembled genomes are also available at NCBI under the umbrella project PRJNA1112097. The software used, with their respective versions and non-default parameters, are described in the methods section (Supplemental material) of this paper. The bioinformatics software and outputs of the analyses described here are also available on the KBase platform. Other supporting data are available in the Supplemental material of this article.

## SUPPLEMENTAL MATERIAL

Supplemental material spectrum.01561-24-s0001.pdf Supplemental material and methods, and Tables S1 to S7.

Supplemental tables spectrum.01561-24-s0002.xlsx Tables S8 to S10.

ASM does not own the copyrights to Supplemental Material that may be linked to, or accessed through, an article. The authors have granted ASM a non-exclusive, world-wide license to publish the Supplemental Material files. Please contact the corresponding author directly for reuse.
