# Supplementary material for: Linking soil microbial genomic features to forest-to-pasture conversion in the Amazon
Source: Microbiol Spectr. 2025 Mar 5;13(4):e01561-24. doi: 10.1128/spectrum.01561-24 (PMC11960080; doi:10.1128/spectrum.01561-24)
Supplement: Supplemental material — and methods, and Tables S1 to S7. [file spectrum.01561-24-s0001.pdf]

SUPPLEMENTARY INFORMATION

Linking soil microbial genomic features to forest-to-pasture conversion in the Amazon

Venturini et al. 2025

Supplementary text

Material and methods

Soil sampling and metagenomic sequencing

The study was conducted in three primary forests (FP2, FP4, and FP5) of the Tapajós National Forest and three active cattle pastures (P2, P3, and P4) nearby, in the state of Pará, Eastern Amazon, Brazil, during dry (November 2015) and rainy (May 2016) seasons. At each site, following the removal of the litter layer, soil samples from 0 to 10 cm depth were collected in a 200-m transect composed of three sampling points, separated by 100 m each (Supplementary Table 1). Soil samples were DNA-extracted in duplicate using the PowerLyzer PowerSoil DNA Isolation Kit (QIAGEN GmbH, Hilden, Germany), following the protocol optimized by Venturini et al. (2020). DNA samples were quantified on a Nanodrop 2000c spectrophotometer (Thermo Fisher Scientific, Inc., Waltham, MA, USA) and shotgun metagenomic sequenced (2 × 150 bp) on an Illumina HiSeq platform (Illumina, Inc., San Diego, CA, USA) at Novogene Co., Ltd. (Beijing, China), using the NEBNext Ultra II DNA Library Prep Kit for Illumina (New England Biolabs, Inc., Ipswich, MA, USA) for library construction.

Quality control, assembly, and binning of reads

Shotgun metagenomic sequences were imported into the KBase platform (Arkin et al., 2018) as paired-end reads, quality-evaluated using FastQC v0.11.9 (Andrews, 2010), trimmed and filtered using Trimmomatic v0.36 (adapters, TruSeq3-PE-2; sliding window minimum quality, 20; head crop length, 10; minimum read length, 100) (Bolger et al., 2014), and re-evaluated using FastQC (Andrews, 2010).

The remaining paired-end reads from each site were merged into one object using the app Merge Reads Libraries v1.0.1 (Arkin et al., 2018) and co-assembled using MEGAHIT v1.2.9 (preset, meta-large) (Li et al., 2015). We used a co-assembly approach to maximize the number of MAGs recovered due to the high diversity of Amazonian soils. Co-assemblies were quality-evaluated using QUAST v.4.4 (Gurevich et al., 2013). Contigs were binned using MaxBin2 v2.2.4 (marker set, both 107 and 40 marker genes; minimum contig length, 2000 bp) (Wu et al., 2016), MetaBAT2 v1.7 (minimum contig length, 2000 bp) (Kang et al., 2015), and CONCOCT v1.1 (minimum contig length, 2000 bp) (Alneberg et al., 2014), followed by optimization by DAS Tool v1.1.2 (Sieber et al., 2018). MAGs were quality-checked (reference tree, full tree) and filtered (reference tree, full tree; completeness, ≥ 50%; contamination, ≤ 10%) using CheckM v1.0.18 (Parks et al., 2015), considering the Minimum Information about a Metagenome-Assembled Genome (MIMAG) standards for medium-quality drafts (Bowers et al., 2017). The coverage of each MAG was estimated using Bowtie2 v2.3.2 (alignment type preset options, very sensitive) (Langmead and Salzberg, 2012). All statistical analyses were performed in R 4.3.1 (R Core Team, 2023). The Mann-Whitney U test was used to compare genomic features of forest and pasture MAGs. Traits were then correlated (Spearman's rank order correlation coefficient). Plots were generated using webr 0.1.5 (Moon, 2020) and ggplot2 3.4.3 (Wickham, 2016).

Taxonomic classification and functional annotation of MAGs

Medium- and high-quality MAGs were taxonomically classified using GTDB-Tk v2.3.2 (release R08-RS214) (Chaumeil et al., 2019), and genomes with identical taxonomic classification were further compared using the app Compute ANI with FastANI v.0.1.3 (Jain et al., 2018). MAGs were annotated using DRAM v.0.1.2 (minimum contig length, 2000 bp) (Shaffer et al., 2020). Sequences from microTrait environmental bioelement cycling families were searched using a set of Hidden Markov Models (HMMs) (app v.1) (Eddy, 2011; Karaoz and Brodie, 2022; Chivian et al., 2023). Sequences from the dbCAN2 carbohydrate-active enzyme families (CAZy) collection were also searched using a set of Hidden Markov Models (HMMs) (app v.10) (Eddy, 2011; Zhang et al., 2018; Chivian et al., 2023). Significant differences in the functional composition of the forest and pasture communities were tested by permutational multivariate ANOVA (PERMANOVA) on Jaccard dissimilarities using vegan 2.6.4 (Oksanen et al., 2022). Non-metric multidimensional scaling (NMDS) plots based on Jaccard dissimilarities were created using the same package. Venn diagrams of functional traits were generated using eulerr 7.0.0 (Larsson, 2022), and a plot with the profile of microTrait genes (related to the nitrogen, methane, and sulfur cycles) in forest and pasture MAGs was created using ggalluvial 0.12.5 (Brunson and Read, 2023) with MetBrewer 0.3.0 (Mills, 2024). Growth conditions (oxygen, temperature, pH, and salinity) for each MAG were predicted using GenomeSPOT v1.0.1 (Barnum et al., 2024), followed by the Mann–Whitney U test, as previously described.

Results

Quality control, assembly, and binning

The metagenomic sequences had between 33 and 58 million 150-bp long paired-end reads ($total_{mean}$, 48; $forest_{mean}$, 49; $pasture_{mean}$, 47 million) (Supplementary Table 2). After trimming and filtering steps, between 22 and 48 million paired-end reads remained ($total_{mean}$, 37 million; $forest_{mean}$, 39 million; $pasture_{mean}$, 35 million), ranging from 100 to 140 bp. Remaining paired-end reads from each site, regardless of the season, were merged, resulting in samples from 187 to 253 million paired-end reads (forest: FP2, 253; FP4, 244; FP5, 197; pasture: P2, 187; P3, 207; P4, 232 million). Assemblies had from 218,238 to 328,152 contigs ($forest_{mean}$, 289,502; $pasture_{mean}$, 254,880), largest contig between 120,589 and 272,602 bp ($forest_{mean}$, 179,489; $pasture_{mean}$, 251,246), N50 between 3,283 and 4,234 bp ($forest_{mean}$, 3,400; $pasture_{mean}$, 4,047), L50 between 55,398 and 97,902 bp ($forest_{mean}$, 86,459; $pasture_{mean}$, 62,520), and GC content between 62% and 67% ($forest_{mean}$, 63%; $pasture_{mean}$, 67%) (Supplementary Table 3). Following contigs binning and optimization, we recovered 115 MAGs (forest: FP2, 24; FP4, 20; FP5, 8; pasture: P2, 29; P3, 21; P4, 13).

References

1. Alneberg J, Bjarnason BS, de Bruijn I, Schirmer M, Quick J, Ijaz UZ, et al. Binning metagenomic contigs by coverage and composition. Nat Methods. 2014; 11(11):1144–6.
2. Andrews S. FastQC: A quality control tool for high throughput sequence data. 2010. http://www.bioinformatics.babraham.ac.uk/ projects/fastqc.
3. Arkin AP, Cottingham RW, Henry CS, Harris NL, Stevens RL, Maslov S, et al. KBase: The United States Department of Energy Systems Biology Knowledgebase. Nat Biotechnol. 2018; 36(7):566–9.
4. Barnum TP, Crits-Christoph A, Molla M, Carini P, Lee HH, Ostrov N. Predicting microbial growth conditions from amino acid composition. bioRxiv. 2024; 2024.03.22.586313.

5. Bolger AM, Lohse M, Usadel B. Trimmomatic: a flexible trimmer for Illumina sequence data. Bioinformatics. 2014; 30(15):2114–20.
6. Bowers RM, Kyrpides NC, Stepanauskas R, Harmon-Smith M, Doud D, Reddy TBK, et al. Minimum information about a single amplified genome (MISAG) and a metagenome-assembled genome (MIMAG) of bacteria and archaea. Nat Biotechnol. 2017; 35(8):725–31.
7. Brunson JC, Read QD. ggalluvial: Alluvial Plots in 'ggplot2'. R package version 0.12.5. 2023. http://corybrunson.github.io/ggalluvial/.
8. Chaumeil PA, Mussig AJ, Hugenholtz P, Parks DH. GTDB-Tk: a toolkit to classify genomes with the Genome Taxonomy Database. Hancock J, editor. Bioinformatics. 2019; btz848.
9. Chivian D, Jungbluth SP, Dehal PS, Wood-Charlson EM, Canon RS, Allen BH, et al. Metagenome-assembled genome extraction and analysis from microbiomes using KBase. Nat Protoc. 2023; 18(1):208–38.
10. Eddy SR. Accelerated Profile HMM Searches. Pearson WR, editor. PLoS Comput Biol. 2011; 7(10):e1002195.
11. Gurevich A, Saveliev V, Vyahhi N, Tesler G. QUAST: quality assessment tool for genome assemblies. Bioinformatics. 2013; 29(8):1072–5.
12. Jain C, Rodriguez-R LM, Phillippy AM, Konstantinidis KT, Aluru S. High throughput ANI analysis of 90K prokaryotic genomes reveals clear species boundaries. Nat Commun. 2018; 9(1):5114.
13. Kang DD, Froula J, Egan R, Wang Z. MetaBAT, an efficient tool for accurately reconstructing single genomes from complex microbial communities. PeerJ. 2015; 3:e1165.
14. Karaoz U, Brodie EL. microTrait: A toolset for a trait-based representation of microbial genomes. Front Bioinform. 2022; 2:918853.
15. Langmead B, Salzberg SL. Fast gapped-read alignment with Bowtie 2. Nat Methods. 2012; 9(4), 357-9.
16. Larsson J. eulerr: Area-Proportional Euler and Venn Diagrams with Ellipses. R package version 7.0.0. 2022. https://CRAN.R-project.org/package=eulerr.
17. Li D, Liu CM, Luo R, Sadakane K, Lam TW. MEGAHIT: an ultra-fast single-node solution for large and complex metagenomics assembly via succinct de Bruijn graph. Bioinformatics. 2015; 31(10):1674–6.
18. Mills BR. MetBrewer: Color Palettes Inspired by Works at the Metropolitan Museum of Art. R package version 0.3.0. 2024. https://github.com/BlakeRMills/MetBrewer.
19. Moon K. webr: Data and Functions for Web-Based Analysis. R package version 0.1.5. 2020. https://CRAN.R-project.org/package=webr.
20. Oksanen J, Simpson G, Blanchet F, Kindt R, Legendre P, Minchin P, et al. vegan: Community Ecology Package. R package version 2.6-4. 2022. https://CRAN.R-project.org/package=vegan.
21. Parks DH, Imelfort M, Skennerton CT, Hugenholtz P, Tyson GW. CheckM: Assessing the quality of microbial genomes recovered from isolates, single cells, and metagenomes. Genome Res. 2015; 25:1043–55.
22. R Core Team. R: A Language and Environment for Statistical Computing. R Foundation for Statistical Computing, Vienna, Austria. 2023. https://www.R-project.org.
23. Shaffer M, Borton MA, McGivern BB, Zayed AA, La Rosa SL, Solden LM, et al. DRAM for distilling microbial metabolism to automate the curation of microbiome function. Nucleic Acids Res. 2020; 48(16):8883–900.

24. Sieber CMK, Probst AJ, Sharrar A, Thomas BC, Hess M, Tringe SG, et al. Recovery of genomes from metagenomes via a dereplication, aggregation and scoring strategy. Nat Microbiol. 2018; 3(7):836–43.
25. Venturini AM, Nakamura FM, Gontijo JB, da França AG, Yoshiura CA, Mandro JA, et al. Robust DNA protocols for tropical soils. Heliyon. 2020; 6(5):e03830.
26. Wickham H. ggplot2: Elegant Graphics for Data Analysis. Springer International Publishing, Cham, 2016.
27. Wu YW, Simmons BA, Singer SW. MaxBin 2.0: an automated binning algorithm to recover genomes from multiple metagenomic datasets. Bioinformatics. 2016; 32(4):605–7.
28. Zhang H, Yohe T, Huang L, Entwistle S, Wu P, Yang Z, et al. dbCAN2: a meta server for automated carbohydrate-active enzyme annotation. Nucleic Acids Res. 2018; 46(W1):W95–101.

Supplementary tables

Material and methods

Supplementary Table S1. Geographical coordinates and elevation of the soil sampling points of the forest and pasture sites.

Land-use	Site	Sampling point	Geographical coordinates	Elevation (m)
		1	2°51'19.6"S 54°57'30.1"W	205
	FP2	3	2°51'18.4"S 54°57'27.2"W	208
		5	2°51'17.4"S 54°57'23.8"W	207
		1	3°01'08.3"S 55°00'23.3"W	104
Forest	FP4	3	3°01'07.4"S 55°00'20.1"W	111
		5	3°01'07.4"S 55°00'17.0"W	119
		1	3°17'44.4"S 54°57'46.7"W	132
	FP5	3	3°17'47.2"S 54°57'48.4"W	132
		5	3°17'49.6"S 54°57'50.6"W	131
		1	3°18'46.7"S 54°54'34.8"W	123
	P2	3	3°18'49.9"S 54°54'35.1"W	133
		5	3°18'53.1"S 54°54'34.7"W	134
		1	3°07'49.8"S 54°57'28.5"W	100
Pasture	P3	3	3°07'52.9"S 54°57'28.1"W	101
		5	3°07'56.0"S 54°57'27.5"W	103
		1	3°07'44.9"S 54°57'15.5"W	99
	P4	3	3°07'42.7"S 54°57'13.0"W	100
		5	3°07'39.5"S 54°57'11.8"W	98

Results

Supplementary Table S2. Number of paired-end reads and length of each metagenomic sample before (Pre-QC) and after (Post-QC) quality control.

	**Metadata**				**Pre-QC**		**Post-QC**	
**ID**	**Land use**	**Season**	**Site**	**Point**	**No. PE reads**	**Length (bp)**	**No. PE reads**	**Length (bp)**
C1	Forest	Dry	FP2	1	50,391,249	150	41,958,340	100 - 140
C2	Forest	Dry	FP2	3	55,700,040	150	47,310,700	100 - 140
C3	Forest	Dry	FP2	5	48,294,413	150	36,151,601	100 - 140
C4	Forest	Dry	FP4	1	54,620,913	150	44,259,551	100 - 140
C5	Forest	Dry	FP4	3	57,458,707	150	47,995,953	100 - 140
C6	Forest	Dry	FP4	5	50,682,023	150	42,249,644	100 - 140
C7	Forest	Dry	FP5	1	51,968,207	150	42,289,082	100 - 140
C8	Forest	Dry	FP5	3	48,194,355	150	39,757,098	100 - 140
C9	Forest	Dry	FP5	5	33,069,369	150	22,286,439	100 - 140
C10	Pasture	Dry	P2	1	36,877,677	150	25,502,175	100 - 140
C11	Pasture	Dry	P2	3	37,136,069	150	25,643,651	100 - 140
C12	Pasture	Dry	P2	5	48,873,529	150	33,638,296	100 - 140
C13	Pasture	Dry	P3	1	45,616,386	150	30,959,657	100 - 140
C14	Pasture	Dry	P3	3	41,199,241	150	26,549,681	100 - 140
C15	Pasture	Dry	P3	5	48,413,365	150	40,300,773	100 - 140
C16	Pasture	Dry	P4	1	50,104,828	150	41,212,744	100 - 140
C17	Pasture	Dry	P4	3	57,565,627	150	48,065,127	100 - 140
C18	Pasture	Dry	P4	5	46,697,931	150	39,301,361	100 - 140
C19	Forest	Rainy	FP2	1	44,907,671	150	38,033,893	100 - 140
C20	Forest	Rainy	FP2	3	55,608,768	150	46,972,660	100 - 140
C21	Forest	Rainy	FP2	5	50,026,199	150	42,786,590	100 - 140
C22	Forest	Rainy	FP4	1	56,713,568	150	47,510,378	100 - 140
C23	Forest	Rainy	FP4	3	43,622,933	150	31,475,510	100 - 140
C24	Forest	Rainy	FP4	5	42,746,571	150	30,772,832	100 - 140
C25	Forest	Rainy	FP5	1	40,431,173	150	30,188,317	100 - 140
C26	Forest	Rainy	FP5	3	41,515,485	150	28,237,595	100 - 140
C27	Forest	Rainy	FP5	5	48,840,395	150	34,503,674	100 - 140
C28	Pasture	Rainy	P2	1	42,652,101	150	31,338,537	100 - 140
C29	Pasture	Rainy	P2	3	45,720,045	150	33,076,097	100 - 140
C30	Pasture	Rainy	P2	5	54,221,253	150	38,262,432	100 - 140
C31	Pasture	Rainy	P3	1	51,984,014	150	38,321,864	100 - 140
C32	Pasture	Rainy	P3	3	49,373,901	150	35,915,715	100 - 140
C33	Pasture	Rainy	P3	5	46,267,199	150	34,452,674	100 - 140
C34	Pasture	Rainy	P4	1	43,222,163	150	32,125,759	100 - 140
C35	Pasture	Rainy	P4	3	46,610,016	150	35,654,571	100 - 140
C36	Pasture	Rainy	P4	5	48,285,554	150	35,900,597	100 - 140

Supplementary Table S3. Quality assessment (QUAST statistics) of forest and pasture soil co-assemblies.

**QUAST statistics**	**Forest_FP2**	**Forest_FP4**	**Forest_FP5**	**Pasture_P2**	**Pasture_P3**	**Pasture_P4**
No. contigs	322,116	328,152	218,238	239,413	239,213	286,013
No. contigs (>= 2000 bp)	322,116	328,152	218,238	239,413	239,213	286,013
No. contigs (>= 10000 bp)	8,562	8,437	4,604	11,082	12,032	11,511
No. contigs (>= 100000 bp)	23	3	1	25	74	52
No. contigs (>= 1000000 bp)	0	0	0	0	0	0
Largest contig	272,602	120,589	145,277	262,885	258,024	232,829
Total length	1,152,641,481	1,158,797,239	745,258,552	956,562,285	995,870,085	1,122,819,180
Total length (>= 2000 bp)	1,152,641,481	1,158,797,239	745,258,552	956,562,285	995,870,085	1,122,819,180
Total length (>= 10000 bp)	142,420,285	128,950,984	72,452,710	195,134,926	238,114,227	222,866,033
Total length (>= 100000 bp)	3,565,935	345,379	145,277	3,331,162	9,810,239	6,709,104
Total length (>= 1000000 bp)	0	0	0	0	0	0
N50	3,478	3,439	3,283	4,030	4,234	3,876
N75	2,522	2,511	2,462	2,660	2,705	2,621
L50	94,136	97,902	67,338	59,944	55,398	72,219
L75	192,835	197,917	133,731	134,605	130,860	162,161
GC (%)	63	63	62	67	67	66
No. Ns	0	0	0	0	0	0

Supplementary Table S4. Detailed information (completeness, contamination, genome size, number of contigs, longest contig, mean contig length, N50, GC content, number of ambiguous bases and predicted coding sequences, coding density, and coverage) of forest and pasture soil MAGs.

MAG	Land use	Site	Completeness (%)	Contamination (%)	Genome size (bp)	No. contigs	Longest contig (bp)	Mean contig length (bp)	N50	GC (%)	No. ambiguous bases	No. predicted coding sequences (CDS)	Coding density (%)	Coverage (x)
Bin.001.fastaField_forest_FP2	Forest	FP2	65.5	0.0	1,835,222	226	40,376	8,120	11,589	68.6	0	1,934	91.7	16
Bin.003.fastaField_forest_FP2	Forest	FP2	78.6	5.1	2,303,042	333	52,727	6,916	9,260	59.8	0	2,484	93.0	12
Bin.006.fastaField_forest_FP2	Forest	FP2	80.1	1.8	4,443,883	408	50,487	10,892	15,162	66.4	0	4,698	93.2	14
Bin.009.fastaField_forest_FP2	Forest	FP2	86.4	1.9	1,042,421	37	100,165	28,174	42,100	40.8	0	1,155	79.4	13
Bin.010.fastaField_forest_FP2	Forest	FP2	86.6	6.0	8,104,940	1,235	63,758	6,563	7,866	56.4	0	8,161	82.2	25
Bin.014.fastaField_forest_FP2	Forest	FP2	69.5	1.0	3,296,092	99	272,602	33,294	108,940	58.9	0	3,488	85.7	28
Bin.016.fastaField_forest_FP2	Forest	FP2	67.1	4.5	2,839,147	480	37,253	5,915	7,258	60.8	0	2,761	90.3	21
Bin.019.fastaField_forest_FP2	Forest	FP2	80.0	1.7	2,254,515	208	96,073	10,839	15,498	72.7	0	2,444	94.3	17
Bin.022.fastaField_forest_FP2	Forest	FP2	78.2	2.9	1,170,067	65	67,554	18,001	34,347	41.0	0	1,358	81.4	19
Bin.023.fastaField_forest_FP2	Forest	FP2	87.2	8.5	3,013,436	397	72,073	7,591	11,225	72.4	0	3,283	90.5	39
Bin.024.fastaField_forest_FP2	Forest	FP2	85.9	6.0	2,039,572	343	43,768	5,946	7,281	63.1	0	2,243	95.0	33
Bin.001.fastaField_forest_FP4	Forest	FP4	64.3	0.9	1,895,143	310	23,475	6,113	6,684	56.6	0	2,034	94.2	8
Bin.002.fastaField_forest_FP4	Forest	FP4	83.7	2.8	2,390,938	410	29,020	5,832	7,253	59.4	0	2,664	84.6	14
Bin.004.fastaField_forest_FP4	Forest	FP4	94.6	3.8	3,460,958	419	39,515	8,260	11,387	54.2	0	3,795	87.6	10
Bin.005.fastaField_forest_FP4	Forest	FP4	80.4	4.8	2,001,731	400	28,041	5,004	5,832	63.0	0	2,260	94.7	22
Bin.008.fastaField_forest_FP4	Forest	FP4	70.8	1.7	4,116,342	717	25,887	5,741	6,367	57.0	0	4,036	84.3	28
Bin.010.fastaField_forest_FP4	Forest	FP4	94.0	3.6	2,783,438	226	113,643	12,316	18,654	67.8	0	2,826	87.9	34
Bin.012.fastaField_forest_FP4	Forest	FP4	69.6	6.0	4,823,373	1,378	16,860	3,500	3,526	38.9	0	6,803	69.8	13
Bin.013.fastaField_forest_FP4	Forest	FP4	57.6	7.4	1,990,159	540	16,875	3,685	3,806	60.0	0	2,349	93.2	7
Bin.015.fastaField_forest_FP4	Forest	FP4	72.7	6.0	3,027,851	556	36,493	5,446	6,426	56.3	0	3,144	92.4	29
Bin.016.fastaField_forest_FP4	Forest	FP4	77.9	5.1	6,757,100	852	37,159	7,931	9,363	64.8	0	6,532	87.4	9
Bin.017.fastaField_forest_FP4	Forest	FP4	79.9	4.4	3,700,804	525	26,419	7,049	8,145	66.8	0	3,622	85.4	10
Bin.001.fastaField_forest_FP5	Forest	FP5	59.4	8.9	4,157,900	981	52,538	4,238	4,673	59.8	0	4,509	85.4	99
Bin.002.fastaField_forest_FP5	Forest	FP5	74.2	5.6	2,011,784	494	15,855	4,072	4,356	69.1	0	2,308	92.6	11
Bin.003.fastaField_forest_FP5	Forest	FP5	73.1	3.8	2,609,634	145	93,163	17,997	26,906	69.9	0	2,677	92.1	18
Bin.005.fastaField_forest_FP5	Forest	FP5	78.1	4.6	2,103,160	462	21,568	4,552	5,055	59.5	0	2,386	85.1	9
Bin.002.fastaField_pasture_P2	Pasture	P2	94.0	3.5	5,663,896	439	141,275	12,902	19,705	72.1	0	5,480	90.5	24

Bin.004.fastaField_pasture_P2	Pasture	P2	87.4	6.5	3,692,752	508	38,439	7,269	9,573	65.0	0	3,655	86.3	10
Bin.006.fastaField_pasture_P2	Pasture	P2	57.0	3.0	3,679,344	674	30,366	5,459	6,641	67.8	0	3,954	91.5	17
Bin.007.fastaField_pasture_P2	Pasture	P2	77.1	2.2	2,538,400	507	20,560	5,007	5,695	71.5	0	2,799	92.3	9
Bin.008.fastaField_pasture_P2	Pasture	P2	78.9	8.5	3,690,378	857	30,022	4,306	5,040	63.6	0	3,815	90.1	7
Bin.009.fastaField_pasture_P2	Pasture	P2	85.3	7.1	3,630,813	358	74,742	10,142	13,619	69.0	0	3,841	90.0	45
Bin.011.fastaField_pasture_P2	Pasture	P2	92.3	2.1	3,975,112	193	142,705	20,596	33,658	69.7	0	4,024	92.9	16
Bin.012.fastaField_pasture_P2	Pasture	P2	74.0	9.0	3,487,761	265	105,427	13,161	19,632	69.5	0	3,588	91.5	37
Bin.013.fastaField_pasture_P2	Pasture	P2	99.2	1.9	3,189,021	63	262,885	50,619	88,649	67.0	0	3,364	93.8	33
Bin.014.fastaField_pasture_P2	Pasture	P2	85.6	1.6	4,222,578	480	52,816	8,797	11,663	67.2	0	4,328	92.0	23
Bin.016.fastaField_pasture_P2	Pasture	P2	80.3	7.4	3,789,146	338	67,073	11,210	15,891	71.6	0	4,080	93.7	40
Bin.017.fastaField_pasture_P2	Pasture	P2	82.5	2.0	4,746,555	610	45,945	7,781	10,107	68.4	0	4,968	91.6	32
Bin.018.fastaField_pasture_P2	Pasture	P2	96.6	9.4	4,123,077	380	179,426	10,850	21,899	38.4	0	5,301	69.5	19
Bin.019.fastaField_pasture_P2	Pasture	P2	67.7	5.6	2,711,864	474	26,316	5,721	6,630	61.7	0	2,924	88.3	60
Bin.020.fastaField_pasture_P2	Pasture	P2	55.9	3.0	2,503,745	692	12,452	3,618	3,794	64.6	0	2,767	87.7	9
Bin.022.fastaField_pasture_P2	Pasture	P2	61.5	0.2	3,617,304	234	107,563	15,459	23,374	56.3	0	3,322	90.1	15
Bin.023.fastaField_pasture_P2	Pasture	P2	55.8	2.4	1,782,746	302	18,961	5,903	6,225	53.7	0	1,886	88.9	13
Bin.024.fastaField_pasture_P2	Pasture	P2	79.9	6.7	2,918,176	314	26,477	9,294	11,996	53.8	0	2,921	91.0	16
Bin.026.fastaField_pasture_P2	Pasture	P2	88.6	2.1	2,973,398	262	71,013	11,349	17,703	70.5	0	3,030	92.7	18
Bin.028.fastaField_pasture_P2	Pasture	P2	92.0	6.6	6,923,874	599	63,547	11,559	14,435	62.6	0	6,166	91.0	9
Bin.001.fastaField_pasture_P3	Pasture	P3	90.0	2.8	7,262,660	635	54,554	11,437	14,952	70.5	0	7,235	91.8	12
Bin.002.fastaField_pasture_P3	Pasture	P3	65.2	1.7	1,731,873	275	25,743	6,298	6,688	53.7	0	1,814	89.2	8
Bin.004.fastaField_pasture_P3	Pasture	P3	92.7	6.7	7,162,282	650	117,972	11,019	24,113	66.6	0	7,357	89.1	17
Bin.006.fastaField_pasture_P3	Pasture	P3	64.7	2.0	1,949,427	150	42,831	12,996	14,039	70.7	0	2,177	94.0	61
Bin.007.fastaField_pasture_P3	Pasture	P3	86.4	4.1	3,221,267	587	35,880	5,488	6,498	67.3	0	3,664	92.7	13
Bin.008.fastaField_pasture_P3	Pasture	P3	96.3	1.5	4,161,081	176	203,727	23,643	40,183	36.2	0	4,806	68.8	16
Bin.009.fastaField_pasture_P3	Pasture	P3	79.7	4.2	3,134,645	600	27,567	5,224	6,317	61.7	0	3,412	88.2	69
Bin.010.fastaField_pasture_P3	Pasture	P3	91.9	3.0	2,841,406	191	93,290	14,876	25,211	62.5	0	3,012	91.0	19
Bin.011.fastaField_pasture_P3	Pasture	P3	64.0	4.6	2,355,054	616	16,755	3,823	3,972	59.4	0	2,584	90.7	6
Bin.013.fastaField_pasture_P3	Pasture	P3	88.0	9.3	4,222,307	227	94,929	18,600	27,089	71.5	0	4,449	94.0	80
Bin.014.fastaField_pasture_P3	Pasture	P3	99.7	2.8	4,300,823	183	209,582	23,502	40,497	69.8	0	4,114	91.6	85
Bin.016.fastaField_pasture_P3	Pasture	P3	88.7	3.3	4,678,543	155	196,655	30,184	75,301	64.0	0	4,553	88.4	18
Bin.017.fastaField_pasture_P3	Pasture	P3	99.7	5.1	5,796,481	190	258,024	30,508	61,293	67.8	0	5,556	86.2	36
Bin.020.fastaField_pasture_P3	Pasture	P3	90.6	0.9	3,581,002	115	129,840	31,139	45,065	69.8	0	3,450	92.6	23
Bin.021.fastaField_pasture_P3	Pasture	P3	86.5	3.1	3,732,017	100	200,614	37,320	80,911	71.2	0	3,494	90.7	31

Bin.001.fastaField_pasture_P4	Pasture	P4	94.1	5.1	3,935,357	241	101,742	16,329	28,626	70.5	0	3,860	88.9	24
Bin.002.fastaField_pasture_P4	Pasture	P4	94.3	0.5	5,991,177	336	93,322	17,831	25,954	63.3	0	6,052	90.1	11
Bin.003.fastaField_pasture_P4	Pasture	P4	78.4	6.4	3,779,530	592	57,147	6,384	8,035	71.2	0	4,076	93.4	49
Bin.004.fastaField_pasture_P4	Pasture	P4	69.0	5.8	2,726,193	596	15,937	4,574	5,149	65.3	0	2,921	86.3	11
Bin.008.fastaField_pasture_P4	Pasture	P4	94.4	3.0	6,849,160	296	167,003	23,139	36,847	65.9	0	6,605	88.3	16
Bin.010.fastaField_pasture_P4	Pasture	P4	87.5	6.0	3,934,026	449	57,801	8,762	11,524	66.9	0	4,266	91.7	40
Bin.011.fastaField_pasture_P4	Pasture	P4	88.3	5.7	4,829,382	276	98,104	17,498	26,862	71.2	0	4,870	92.1	39
Bin.012.fastaField_pasture_P4	Pasture	P4	84.4	5.1	3,816,424	374	73,805	10,204	17,160	67.5	0	3,960	89.4	24

Supplementary Table S5. Number of 5S, 16S, and 23S rRNA genes and tRNAs found in forest and pasture soil MAGs.

MAG	Land use	Site	No. of 5S rRNAs	No. of 16S rRNAs	No. of 23S rRNAs	No. of tRNAs
Bin.001.fastaField_forest_FP2	Forest	FP2	0	0	0	10
Bin.003.fastaField_forest_FP2	Forest	FP2	0	0	0	22
Bin.006.fastaField_forest_FP2	Forest	FP2	1	0	0	34
Bin.009.fastaField_forest_FP2	Forest	FP2	0	1	0	32
Bin.010.fastaField_forest_FP2	Forest	FP2	1	0	0	50
Bin.014.fastaField_forest_FP2	Forest	FP2	0	0	0	29
Bin.016.fastaField_forest_FP2	Forest	FP2	0	0	0	7
Bin.019.fastaField_forest_FP2	Forest	FP2	0	0	0	38
Bin.022.fastaField_forest_FP2	Forest	FP2	2	0	0	39
Bin.023.fastaField_forest_FP2	Forest	FP2	2	1	0	33
Bin.024.fastaField_forest_FP2	Forest	FP2	1	0	0	23
Bin.001.fastaField_forest_FP4	Forest	FP4	0	0	0	26
Bin.002.fastaField_forest_FP4	Forest	FP4	0	0	0	23
Bin.004.fastaField_forest_FP4	Forest	FP4	1	1	0	40
Bin.005.fastaField_forest_FP4	Forest	FP4	1	0	0	28
Bin.008.fastaField_forest_FP4	Forest	FP4	1	0	0	7
Bin.010.fastaField_forest_FP4	Forest	FP4	1	0	0	48
Bin.012.fastaField_forest_FP4	Forest	FP4	1	0	0	39
Bin.013.fastaField_forest_FP4	Forest	FP4	0	0	0	20
Bin.015.fastaField_forest_FP4	Forest	FP4	0	1	0	22
Bin.016.fastaField_forest_FP4	Forest	FP4	0	0	0	25
Bin.017.fastaField_forest_FP4	Forest	FP4	0	0	0	14
Bin.001.fastaField_forest_FP5	Forest	FP5	1	0	0	20
Bin.002.fastaField_forest_FP5	Forest	FP5	1	0	0	19
Bin.003.fastaField_forest_FP5	Forest	FP5	0	0	0	25
Bin.005.fastaField_forest_FP5	Forest	FP5	0	0	0	28
Bin.002.fastaField_pasture_P2	Pasture	P2	0	1	0	30
Bin.004.fastaField_pasture_P2	Pasture	P2	0	0	0	13
Bin.006.fastaField_pasture_P2	Pasture	P2	0	0	0	9
Bin.007.fastaField_pasture_P2	Pasture	P2	0	0	0	26
Bin.008.fastaField_pasture_P2	Pasture	P2	0	0	0	21
Bin.009.fastaField_pasture_P2	Pasture	P2	0	0	0	41
Bin.011.fastaField_pasture_P2	Pasture	P2	0	1	0	38
Bin.012.fastaField_pasture_P2	Pasture	P2	1	0	0	39
Bin.013.fastaField_pasture_P2	Pasture	P2	1	1	0	73
Bin.014.fastaField_pasture_P2	Pasture	P2	1	0	0	28
Bin.016.fastaField_pasture_P2	Pasture	P2	1	0	0	46
Bin.017.fastaField_pasture_P2	Pasture	P2	1	0	0	39
Bin.018.fastaField_pasture_P2	Pasture	P2	2	0	0	71
Bin.019.fastaField_pasture_P2	Pasture	P2	0	0	0	10
Bin.020.fastaField_pasture_P2	Pasture	P2	0	0	0	16
Bin.022.fastaField_pasture_P2	Pasture	P2	1	0	0	24
Bin.023.fastaField_pasture_P2	Pasture	P2	0	0	0	7
Bin.024.fastaField_pasture_P2	Pasture	P2	1	0	0	39
Bin.026.fastaField_pasture_P2	Pasture	P2	0	0	0	28
Bin.028.fastaField_pasture_P2	Pasture	P2	0	0	0	44
Bin.001.fastaField_pasture_P3	Pasture	P3	1	0	0	80

Bin.002.fastaField_pasture_P3	Pasture	P3	0	0	0	12
Bin.004.fastaField_pasture_P3	Pasture	P3	0	0	0	95
Bin.006.fastaField_pasture_P3	Pasture	P3	0	0	0	28
Bin.007.fastaField_pasture_P3	Pasture	P3	0	0	0	35
Bin.008.fastaField_pasture_P3	Pasture	P3	1	1	0	40
Bin.009.fastaField_pasture_P3	Pasture	P3	1	1	0	15
Bin.010.fastaField_pasture_P3	Pasture	P3	2	0	1	49
Bin.011.fastaField_pasture_P3	Pasture	P3	1	0	0	26
Bin.013.fastaField_pasture_P3	Pasture	P3	2	0	1	50
Bin.014.fastaField_pasture_P3	Pasture	P3	2	0	0	41
Bin.016.fastaField_pasture_P3	Pasture	P3	1	0	0	42
Bin.017.fastaField_pasture_P3	Pasture	P3	2	0	0	55
Bin.020.fastaField_pasture_P3	Pasture	P3	1	0	1	39
Bin.021.fastaField_pasture_P3	Pasture	P3	0	0	0	24
Bin.001.fastaField_pasture_P4	Pasture	P4	1	0	0	53
Bin.002.fastaField_pasture_P4	Pasture	P4	0	0	0	44
Bin.003.fastaField_pasture_P4	Pasture	P4	0	0	0	23
Bin.004.fastaField_pasture_P4	Pasture	P4	0	0	0	6
Bin.008.fastaField_pasture_P4	Pasture	P4	1	1	0	46
Bin.010.fastaField_pasture_P4	Pasture	P4	1	0	0	36
Bin.011.fastaField_pasture_P4	Pasture	P4	1	0	0	40
Bin.012.fastaField_pasture_P4	Pasture	P4	0	0	0	55

Supplementary Table S6. Genome taxonomic database (GTDB) classification of forest and pasture soil MAGs.

MAG	Land use	Site	GTDB classification						
			Domain	Phylum	Class	Order	Family	Genus	Species
Bin.001.fastaField_forest_FP2	Forest	FP2	Bacteria	Actinomycetota	Thermoleophilia	Solirubrobacterales	Solirubrobacteraceae	Palsa-465	-
Bin.003.fastaField_forest_FP2	Forest	FP2	Bacteria	Eremiobacterota	Eremiobacteria	Eremiobacterales	Eremiobacteraceae	JAFANK01	-
Bin.006.fastaField_forest_FP2	Forest	FP2	Bacteria	Pseudomonadota	Alphaproteobacteria	Reyranellales	Reyranellaceae	Reyranella	-
Bin.009.fastaField_forest_FP2	Forest	FP2	Archaea	Thermoproteota	Nitrososphaeria	Nitrososphaerales	Nitrososphaeraceae	Nitrosopolaris	-
Bin.010.fastaField_forest_FP2	Forest	FP2	Bacteria	Acidobacteriota	Terriglobia	Terriglobales	SbA1	Gp1-AA122	-
Bin.014.fastaField_forest_FP2	Forest	FP2	Bacteria	Pseudomonadota	Alphaproteobacteria	Rhizobiales	Xanthobacteraceae	Pseudolabrys	-
Bin.016.fastaField_forest_FP2	Forest	FP2	Bacteria	Acidobacteriota	Terriglobia	Acidoferrales	UBA7541	-	-
Bin.019.fastaField_forest_FP2	Forest	FP2	Bacteria	Actinomycetota	Acidimicrobiia	IMCC26256	PALSA-555	PALSA-555	-
Bin.022.fastaField_forest_FP2	Forest	FP2	Archaea	Thermoproteota	Nitrososphaeria	Nitrososphaerales	Nitrososphaeraceae	Nitrosopolaris	-
Bin.023.fastaField_forest_FP2	Forest	FP2	Bacteria	Actinomycetota	Thermoleophilia	Gaiellales	Gaiellaceae	PALSA-600	-
Bin.024.fastaField_forest_FP2	Forest	FP2	Bacteria	Eremiobacterota	Eremiobacteria	Baltobacterales	Baltobacteraceae	-	-
Bin.001.fastaField_forest_FP4	Forest	FP4	Bacteria	Eremiobacterota	Eremiobacteria	Eremiobacterales	Eremiobacteraceae	JAFANK01	-
Bin.002.fastaField_forest_FP4	Forest	FP4	Bacteria	Pseudomonadota	Alphaproteobacteria	Rhizobiales	Beijerinckiaceae	Methylocella	-
Bin.004.fastaField_forest_FP4	Forest	FP4	Bacteria	Verrucomicrobiota	Verrucomicrobiae	Chthoniobacterales	UBA10450	Udaeobacter	-
Bin.005.fastaField_forest_FP4	Forest	FP4	Bacteria	Eremiobacterota	Eremiobacteria	Baltobacterales	Baltobacteraceae	-	-
Bin.008.fastaField_forest_FP4	Forest	FP4	Bacteria	Acidobacteriota	Terriglobia	Terriglobales	SbA1	Gp1-AA122	-
Bin.010.fastaField_forest_FP4	Forest	FP4	Bacteria	Actinomycetota	Acidimicrobiia	Acidimicrobiales	RAAP-2	Palsa-461	-
Bin.012.fastaField_forest_FP4	Forest	FP4	Archaea	Thermoproteota	Nitrososphaeria	Nitrososphaerales	Nitrososphaeraceae	Nitrosopolaris	-
Bin.013.fastaField_forest_FP4	Forest	FP4	Bacteria	Eremiobacterota	Eremiobacteria	Eremiobacterales	Eremiobacteraceae	JAFANK01	-
Bin.015.fastaField_forest_FP4	Forest	FP4	Bacteria	Acidobacteriota	Terriglobia	20CM-2-55-15	20CM-2-55-15	20CM-2-55-15	-
Bin.016.fastaField_forest_FP4	Forest	FP4	Bacteria	Acidobacteriota	Vicinamibacteria	Vicinamibacterales	UBA2999	Gp6-AA45	-
Bin.017.fastaField_forest_FP4	Forest	FP4	Bacteria	Pseudomonadota	Alphaproteobacteria	Rhizobiales	Hyphomicrobiaceae	AWTP1-13	-
Bin.001.fastaField_forest_FP5	Forest	FP5	Bacteria	Acidobacteriota	Terriglobia	Acidoferrales	UBA7541	Acidoferrum	-
Bin.002.fastaField_forest_FP5	Forest	FP5	Bacteria	Actinomycetota	Acidimicrobiia	Acidimicrobiales	RAAP-2	Bog-756	-
Bin.003.fastaField_forest_FP5	Forest	FP5	Bacteria	Actinomycetota	Acidimicrobiia	Acidimicrobiales	AC-9	AC-9	-
Bin.005.fastaField_forest_FP5	Forest	FP5	Bacteria	Pseudomonadota	Alphaproteobacteria	Rhizobiales	Beijerinckiaceae	Methylocella	-
Bin.002.fastaField_pasture_P2	Pasture	P2	Bacteria	Actinomycetota	Actinomycetia	-	-	-	-
Bin.004.fastaField_pasture_P2	Pasture	P2	Bacteria	Pseudomonadota	Alphaproteobacteria	Rhizobiales	Hyphomicrobiaceae	AWTP1-13	AWTP1-13 sp024281145
Bin.006.fastaField_pasture_P2	Pasture	P2	Bacteria	Actinomycetota	Actinomycetia	Streptosporangiales	Streptosporangiaceae	Chersky-822	-
Bin.007.fastaField_pasture_P2	Pasture	P2	Bacteria	Chloroflexota	Limnocylindria	Limnocylindrales	CSP1-4	CF-46	-

Bin.008.fastaField_pasture_P2	Pasture	P2	Bacteria	Acidobacteriota	Terriglobia	Terriglobales	SbA1	JAMXLI01	JAMXLI01 sp024281115
Bin.009.fastaField_pasture_P2	Pasture	P2	Bacteria	Actinomycetota	Acidimicrobiia	Acidimicrobiales	AC-9	AC-9	-
Bin.011.fastaField_pasture_P2	Pasture	P2	Bacteria	Actinomycetota	Acidimicrobiia	IMCC26256	JAMXLJ01	JAMXLJ01	JAMXLJ01 sp024281095
Bin.012.fastaField_pasture_P2	Pasture	P2	Bacteria	Actinomycetota	Acidimicrobiia	Acidimicrobiales	AC-9	AC-9	-
Bin.013.fastaField_pasture_P2	Pasture	P2	Bacteria	Dormibacterota	Dormibacteria	Dormibacterales	Dormibacteraceae	40CM-4-65-16	40CM-4-65-16 sp024280995
Bin.014.fastaField_pasture_P2	Pasture	P2	Bacteria	Actinomycetota	Actinomycetia	Mycobacteriales	Mycobacteriaceae	Mycobacterium	-
Bin.016.fastaField_pasture_P2	Pasture	P2	Bacteria	Actinomycetota	Thermoleophilia	Gaiellales	Gaiellaceae	-	-
Bin.017.fastaField_pasture_P2	Pasture	P2	Bacteria	Actinomycetota	Actinomycetia	Mycobacteriales	Jatrophihabitantaceae	JAFAWL01	-
Bin.018.fastaField_pasture_P2	Pasture	P2	Archaea	Thermoproteota	Nitrososphaeria	Nitrososphaerales	Nitrososphaeraceae	Nitrosopolaris	Nitrosopolaris sp009898475
Bin.019.fastaField_pasture_P2	Pasture	P2	Bacteria	Pseudomonadota	Alphaproteobacteria	Rhizobiales	Xanthobacteraceae	BOG-931	-
Bin.020.fastaField_pasture_P2	Pasture	P2	Bacteria	Chloroflexota	Ktedonobacteria	Ktedonobacterales	JADMIN01	-	-
Bin.022.fastaField_pasture_P2	Pasture	P2	Bacteria	Acidobacteriota	Terriglobia	Terriglobales	SbA1	-	-
Bin.023.fastaField_pasture_P2	Pasture	P2	Bacteria	Verrucomicrobiota	Verrucomicrobiae	Chthoniobacterales	JAFAMB01	-	-
Bin.024.fastaField_pasture_P2	Pasture	P2	Bacteria	Verrucomicrobiota	Verrucomicrobiae	Chthoniobacterales	UBA10450	Udaeobacter	-
Bin.026.fastaField_pasture_P2	Pasture	P2	Bacteria	Actinomycetota	Acidimicrobiia	Acidimicrobiales	AC-9	AC-9	-
Bin.028.fastaField_pasture_P2	Pasture	P2	Bacteria	Acidobacteriota	Vicinamibacteria	Vicinamibacterales	2-12-FULL-66-21	2-12-FULL-66-21	-
Bin.001.fastaField_pasture_P3	Pasture	P3	Bacteria	Actinomycetota	Actinomycetia	Mycobacteriales	Micromonosporaceae	Rugosimonospora	-
Bin.002.fastaField_pasture_P3	Pasture	P3	Bacteria	Verrucomicrobiota	Verrucomicrobiae	Chthoniobacterales	JAFAMB01	-	-
Bin.004.fastaField_pasture_P3	Pasture	P3	Bacteria	Actinomycetota	Actinomycetia	Streptosporangiales	Streptosporangiaceae	Chersky-822	-
Bin.006.fastaField_pasture_P3	Pasture	P3	Bacteria	Actinomycetota	Thermoleophilia	Gaiellales	Gaiellaceae	13-2-20CM-68-14	-
Bin.007.fastaField_pasture_P3	Pasture	P3	Bacteria	Actinomycetota	Acidimicrobiia	Acidimicrobiales	Palsa-688	-	-
Bin.008.fastaField_pasture_P3	Pasture	P3	Archaea	Thermoproteota	Nitrososphaeria	Nitrososphaerales	Nitrososphaeraceae	TH5896	-
Bin.009.fastaField_pasture_P3	Pasture	P3	Bacteria	Pseudomonadota	Alphaproteobacteria	Rhizobiales	Xanthobacteraceae	BOG-931	-
Bin.010.fastaField_pasture_P3	Pasture	P3	Bacteria	Pseudomonadota	Alphaproteobacteria	Sphingomonadales	Sphingomonadaceae	Sphingomicrobium	-
Bin.011.fastaField_pasture_P3	Pasture	P3	Bacteria	Verrucomicrobiota	Verrucomicrobiae	Chthoniobacterales	UBA10450	-	-
Bin.013.fastaField_pasture_P3	Pasture	P3	Bacteria	Actinomycetota	Thermoleophilia	Gaiellales	Gaiellaceae	-	-
Bin.014.fastaField_pasture_P3	Pasture	P3	Bacteria	Actinomycetota	Actinomycetia	Actinomycetales	Dermatophilaceae	Intrasporangium	-
Bin.016.fastaField_pasture_P3	Pasture	P3	Bacteria	Pseudomonadota	Alphaproteobacteria	Rhizobiales	Xanthobacteraceae	JAFAXD01	-
Bin.017.fastaField_pasture_P3	Pasture	P3	Bacteria	Actinomycetota	Actinomycetia	Mycobacteriales	Pseudonocardiaceae	-	-
Bin.020.fastaField_pasture_P3	Pasture	P3	Bacteria	Actinomycetota	UBA4738	UBA4738	UBA4738	-	-
Bin.021.fastaField_pasture_P3	Pasture	P3	Bacteria	Actinomycetota	Actinomycetia	Streptosporangiales	Streptosporangiaceae	-	-
Bin.001.fastaField_pasture_P4	Pasture	P4	Bacteria	Actinomycetota	Thermoleophilia	Solirubrobacterales	70-9	VAYN01	-
Bin.002.fastaField_pasture_P4	Pasture	P4	Bacteria	Pseudomonadota	Alphaproteobacteria	Acetobacterales	Acetobacteraceae	JAMXLO01	JAMXLO01 sp024280945
Bin.003.fastaField_pasture_P4	Pasture	P4	Bacteria	Actinomycetota	Thermoleophilia	Solirubrobacterales	Solirubrobacteraceae	Palsa-465	-

Bin.004.fastaField_pasture_P4	Pasture	P4	Bacteria	Pseudomonadota	Alphaproteobacteria	Rhizobiales	Hyphomicrobiaceae	AWTP1-13	AWTP1-13 sp024281145
Bin.008.fastaField_pasture_P4	Pasture	P4	Bacteria	Actinomycetota	Actinomycetia	Mycobacteriales	QHCD01	QHCD01	-
Bin.010.fastaField_pasture_P4	Pasture	P4	Bacteria	Actinomycetota	Acidimicrobiia	Acidimicrobiales	Palsa-688	-	-
Bin.011.fastaField_pasture_P4	Pasture	P4	Bacteria	Actinomycetota	Thermoleophilia	Solirubrobacterales	Solirubrobacteraceae	Palsa-465	-
Bin.012.fastaField_pasture_P4	Pasture	P4	Bacteria	Actinomycetota	Acidimicrobiia	Acidimicrobiales	RAAP-2	Bog-473	-

Supplementary Table S7. Pairs of forest and pasture soil MAGs with average nucleotide identity (ANI) equal to or higher than 95%.

Pair (ANI ≥ 95%)	MAG	Land use	Site	GTDB classification						
				Domain	Phylum	Class	Order	Family	Genus	Species
1	Bin.004.fastaField_pasture_P2	Pasture	P2	Bacteria	Pseudomonadota	Alphaproteobacteria	Rhizobiales	Hyphomicrobiaceae	AWTP1-13	AWTP1-13 sp024281145
	Bin.004.fastaField_pasture_P4	Pasture	P4	Bacteria	Pseudomonadota	Alphaproteobacteria	Rhizobiales	Hyphomicrobiaceae	AWTP1-13	AWTP1-13 sp024281145
2	Bin.009.fastaField_forest_FP2	Forest	FP2	Archaea	Thermoproteota	Nitrososphaeria	Nitrososphaerales	Nitrososphaeraceae	Nitrosopolaris	-
	Bin.012.fastaField_forest_FP4	Forest	FP4	Archaea	Thermoproteota	Nitrososphaeria	Nitrososphaerales	Nitrososphaeraceae	Nitrosopolaris	-
3	Bin.010.fastaField_forest_FP2	Forest	FP2	Bacteria	Acidobacteriota	Terriglobia	Terriglobales	SbA1	Gp1-AA122	-
	Bin.008.fastaField_forest_FP4	Forest	FP4	Bacteria	Acidobacteriota	Terriglobia	Terriglobales	SbA1	Gp1-AA122	-
4	Bin.003.fastaField_forest_FP5	Forest	FP5	Bacteria	Actinomycetota	Acidimicrobiia	Acidimicrobiales	AC-9	AC-9	-
	Bin.009.fastaField_pasture_P2	Pasture	P2	Bacteria	Actinomycetota	Acidimicrobiia	Acidimicrobiales	AC-9	AC-9	-
5	Bin.007.fastaField_pasture_P3	Pasture	P3	Bacteria	Actinomycetota	Acidimicrobiia	Acidimicrobiales	Palsa-688	-	-
	Bin.010.fastaField_pasture_P4	Pasture	P4	Bacteria	Actinomycetota	Acidimicrobiia	Acidimicrobiales	Palsa-688	-	-
6	Bin.016.fastaField_pasture_P2	Pasture	P2	Bacteria	Actinomycetota	Thermoleophilia	Gaiellales	Gaiellaceae	-	-
	Bin.013.fastaField_pasture_P3	Pasture	P3	Bacteria	Actinomycetota	Thermoleophilia	Gaiellales	Gaiellaceae	-	-
7	Bin.024.fastaField_forest_FP2	Forest	FP2	Bacteria	Eremiobacterota	Eremiobacteria	Baltobacterales	Baltobacteraceae	-	-
	Bin.005.fastaField_forest_FP4	Forest	FP4	Bacteria	Eremiobacterota	Eremiobacteria	Baltobacterales	Baltobacteraceae	-	-
8	Bin.002.fastaField_forest_FP4	Forest	FP4	Bacteria	Pseudomonadota	Alphaproteobacteria	Rhizobiales	Beijerinckiaceae	Methylocella	-
	Bin.005.fastaField_forest_FP5	Forest	FP5	Bacteria	Pseudomonadota	Alphaproteobacteria	Rhizobiales	Beijerinckiaceae	Methylocella	-
9	Bin.019.fastaField_pasture_P2	Pasture	P2	Bacteria	Pseudomonadota	Alphaproteobacteria	Rhizobiales	Xanthobacteraceae	BOG-931	-
	Bin.009.fastaField_pasture_P3	Pasture	P3	Bacteria	Pseudomonadota	Alphaproteobacteria	Rhizobiales	Xanthobacteraceae	BOG-931	-
10	Bin.023.fastaField_pasture_P2	Pasture	P2	Bacteria	Verrucomicrobiota	Verrucomicrobiae	Chthoniobacterales	JAFAMB01	-	-
	Bin.002.fastaField_pasture_P3	Pasture	P3	Bacteria	Verrucomicrobiota	Verrucomicrobiae	Chthoniobacterales	JAFAMB01	-	-
